# Greater Strength Drives Difference in Power between Sexes in the Conventional Deadlift Exercise

**DOI:** 10.3390/sports4030043

**Published:** 2016-08-05

**Authors:** Margaret T. Jones, Andrew R. Jagim, G. Gregory Haff, Patrick J. Carr, Joel Martin, Jonathan M. Oliver

**Affiliations:** 1 Health and Human Performance, George Mason University, Manassas, VA 20110, USA; mjones15@gmu.edu (M.T.J.); pjcarr540@gmail.com (P.J.C.); jmarti38@gmu.edu (J.M.); 2 Exercise and Sport Science, University of Wisconsin-La Crosse, La Crosse, WI 54601, USA; ajagim@uwlax.edu; 3 Centre for Exercise and Sport Science Research, Edith Cowan University, Joonalup, WA 6027, Australia; g.haff@ecu.edu.au; 4 Kinesiology Department, Texas Christian University, Fort Worth, TX 76129, USA

**Keywords:** gender differences, resistance, velocity, 1-RM deadlift

## Abstract

Limited research exists comparing sex differences in muscular power. The primary purpose of this research was to determine if differences exist in power and velocity in the conventional deadlift (CDL). A secondary purpose was to examine the relationship among power, velocity, strength, and fat free mass (FFM). Eighteen strength trained athletes with ≥1 year CDL experience (women: *n* = 9, 29 ± 2 years, 162.3 ± 1.8 cm, 62 ± 2.4 kg, 23.3 ± 3.2 % body fat (%BF); men: *n* = 9, 29 ± 3 years, 175.6 ± 1.8 cm, 85.5 ± 1.4 kg, 14.8 ± 2.4 %BF), and ≥1.5 one repetition maximum (1-RM) CDL: body mass (BM) ratio (women: 1.6 ± 0.1 1-RM CDL: BM; men: 2.3 ± 0.1 1-RM CDL: BM), performed baseline (body composition, 1-RM CDL) and experimental sessions, in which velocity and power were measured at 30%, 60%, and 90% 1-RM. Repeated measures ANOVA and bivariate correlations were conducted. Men produced higher absolute average and peak power across all loads, but higher average velocity at only 30% 1-RM. When normalized to FFM, men produced higher peak and average power; however, women produced higher peak and average velocities across all loads. FFM and 1-RM were correlated with power. Greater power observed in men is driven by larger muscle mass, which contributes to greater strength.

## 1. Introduction

The ability to generate high power output is a necessary aspect of sport performance [1]. However, the majority of sports require athletes to produce power under varied conditions (e.g., directional change, sprinting). In order to maximize muscular power, a mixed model approach to power training may produce superior results compared to a one-dimensional approach [2,3,4]. In practice, a mixed model approach to power training incorporates exercises performed with low loads at high velocities and high loads at low velocities, thereby affecting a greater portion of the force-velocity relationship compared to a one-dimensional approach in which a smaller portion of the curve is affected [3]. In theory, this approach to training would promote more favorable neuromuscular adaptations with the focus of maximizing an athlete’s power generating capabilities across a broader range of external loads or intensities. Haff and Nimphius [3] reviewed previous strategies that were employed in the design of a mixed model approach to power training, each demonstrating improvement above a one-dimensional approach in men subjects [5,6,7].

While limited research exists comparing sex differences in power training, recent reports suggest that employing different strategies for men and women may be more effective at improving power. For instance, differences have been reported in the load that maximizes power output [8] between men and women [9,10]. Thomas et al. [9] reported a main effect of sex on the optimal load in the squat jump and bench press throw exercises in collegiate soccer athletes. A greater range of loads was reported to have similar power outputs in women compared to men. Further, Garhammer [11] observed that men produce higher power in both absolute and relative terms when examining power output values of elite weightlifters during competition. The results of that analysis and those of a review conducted by Laubach [12], led Garhammer [11] to conclude that women have a lower capacity in slower, strength-oriented lower body activities than in faster, power-oriented lower body activities. From a practical standpoint, those data would suggest that differing strategies might need to be employed when designing a mixed model approach to power training for women. As an example, the time spent devoted to the development of a specific area of the force-velocity relationship (high load-low velocity or low load-high velocity) may need to be adjusted based upon sex. However, to the authors’ knowledge, a direct comparison of such training strategies between sexes has not been conducted. Further, given the results observed by Cormie et al. [13] in which similar short-term improvements in maximal power output were reported in men, who were characterized as weak, after both heavy strength training and ballistic training, differences observed in those aforementioned studies [9,10,11] may not be due solely to sex, but instead differences in absolute strength.

The conventional deadlift is a multi-joint exercise in which a high number of large muscles are recruited [14,15,16] and although traditionally associated with strength training, performing deadlifts at lower loads with higher velocities has been reportedly used for training power in elite athletes [17]. Additionally, conventional deadlift training has been shown to improve rapid torque characteristics in knee extensors and flexors in untrained men and women [18].

Therefore, the purpose of the current investigation was to determine if differences exist between sexes in average power, peak power, and velocity of movement in the conventional deadlift exercise at 30%, 60% and 90% one repetition maximum (1-RM). A secondary purpose was to compare the relationships between power, velocity, strength, and fat free mass.

## 2. Materials and Methods

### 2.1. Subjects

Eighteen athletes (9 men, 9 women) participated in this study completing four testing sessions over four weeks. All subjects voluntarily signed an institutionally approved consent form to participate. The Institutional Review Board for Human Subjects at George Mason University approved all procedures. Severe musculoskeletal injury of the lower body or spinal injuries within 6 months before the study were grounds for exclusion from the study. Subjects were competitive strength-trained athletes with ≥1 year of experience with the conventional deadlift (CDL) exercise, who consistently incorporated the CDL as part of their resistance-training program. Data from baseline demographics and physical characteristics of subjects are included in Table 1. 

### 2.2. Procedures

To determine if differences exist between sexes in power and velocity of movement in the conventional deadlift exercise (CDL), a repeated-measures experimental design was employed. Competitive strength-trained athletes, who used the CDL as a basis of training at differing stages within their respective preparatory training cycle, were recruited to participate. After the baseline session, which included body composition and one-repetition maximum (1-RM) CDL testing, subjects returned to the laboratory on three separate occasions to perform the CDL across specific loads (30%, 60%, 90% 1-RM). Selected loads were identified based upon the congruence of these loads with specificity of training for velocity (30% 1-RM), power (60% 1-RM), and strength (90% 1-RM). Power and velocity were measured and recorded using a linear position transducer and subsequently analyzed. The four testing sessions occurred at the same time of day for each subject. 

### 2.3. Body Composition

Subjects were instructed to drink only water and not to eat or exercise for the preceding two hours. Further, subjects were advised to refrain from any training for the preceding 24 h and no lower body training for the preceding 48 h. Upon arrival to the laboratory, height and body mass were recorded to the nearest 0.01 cm and 0.02 kg, respectively using a stadiometer and digital scale (Bod Pod; Cosmed, Chicago, IL, USA) calibrated according to manufacturer guidelines with subjects bare foot. Body composition was then assessed using air displacement plethysmography (Bod Pod; Cosmed) calibrated according to manufacturer guidelines. Lycra and swim caps were worn during testing and jewelry was removed prior in accordance with standard operating procedures to reduce air displacement. A trained Bod Pod technician performed all testing. Previous studies indicate air displacement plethysmography to be an accurate and reliable means to assess changes in body composition [19]. Body mass and body volume were then used to estimate body fat percentage (%BF) based on the Brozek equation for men and women [20]. Fat free mass (FFM) and fat free mass index (FFMI) were calculated using the following equations.

FFM = Body mass (BM) (kg) × (1 − %BF)

$$\text{FFMI}=\frac{\text{Fat free mass}\;\left(\text{FFM}\right)\;\left(\text{kg}\right)}{{\text{Body height}}^{2}\left(m\right)}$$

### 2.4. One-Repetition Maximum (1-RM)

Following determination of body composition, subjects completed a supervised, standardized, 15-min warm-up prior to determination of 1-RM in the CDL exercise. Subjects then began performing warm-up sets in the CDL. Subjects performed one set of three repetitions followed by forty-five seconds rest (1 × 3 × 50% 1-RM 45 s rest); 1 × 3 × 60% 1-RM 45 s; 2 × 2 × 70% 1-RM 1 min; 1 × 1 × 80% 1-RM 2 min; 1 × 1 × 90% 2.5 min; 1 × 1 × 95% 1-RM 3 min; 100% or greater for 1-RM attempts. Multiple 1-RM attempts were allowed with the 1-RM test concluding when subjects failed to complete a successful lift within 2 attempts. Weightlifting belts and chalk were allowed based upon subject preference. Standard Olympic-style barbells and barbell weights were used (Power Lift, Jefferson, IA, USA). A Certified Strength and Conditioning Specialist© supervised all testing.

### 2.5. Experimental Protocol

After 1-RM testing, subjects returned to the laboratory on three separate occasions to perform the experimental protocol using each of the three randomly assigned loads (30% 1-RM, 60% 1-RM, 90% 1-RM). Each experimental trial was separated by seven days and conducted at the same time of day. On the day of each experimental trial, subjects arrived having refrained from any training or activities outside of daily living for at least 24 h and lower body training for at least 48 h. After a supervised, standardized, 15-min warm-up identical to that performed prior to 1-RM CDL testing, subjects began performing a progression to the desired load for that experimental trial. The progressions to the load to be tested (i.e., 30%, 60% or 90% 1-RM) for the given session were: 30% 1-RM: 3 × 1 @ 30%; 60% 1-RM: 1 × 3 @ 50%, 45 s rest, 3 × 1 @ 60%; 90% 1-RM: 1 × 3 @ 50%, 45 s rest, 1 × 3 @ 60%, 45 s rest, 2 × 2 @ 70%, 1 min rest, 1 × 1 @ 80% 1.5 min rest, 1 × 3 @ 90%. When subjects reached the desired load, three attempts at the load were performed separated by two minutes rest. Subjects were instructed verbally to perform each of the three attempts “as explosively as possible”. This was stated prior to the initiation of each attempt. All attempts were performed to full extension with a verbal command provided once full extension was achieved signaling that the athlete was clear to drop the weighted barbell. Belts and chalk were again allowed based upon subject preference. For each CDL attempt at the specified load, peak power, average power, peak velocity, and average velocity of the weighted barbell were recorded using a linear position transducer (Tendo Fitrodyne, Tendo Sport Machines, Slovak Republic) attached to the right side of the bar in accordance with manufacturer instruction. The reliability of the Tendo unit has been previously reported [21]. Further, the reliability of the Tendo unit used in this study was assessed at each of the selected intensities (30%, 60%, and 90% 1-RM) by comparison of average power among trials. The intra-class correlation coefficient for this comparison was 0.98, 0.99, and 0.96 at 30%, 60%, and 90% 1-RM, respectively. The average of all three attempts was used for statistical analyses.

### 2.6. Statistical Analysis

All data were normally distributed as determined by the Kolomogrov-Smirnov test of normality. Velocity and power data, absolute values and normalized to FFM and FFMI, were assessed by a two factor (sex x load) mixed factorial analysis of variance (ANOVA). Least Squares Difference (LSD) post hoc analyses were performed when a significant finding (*p* ≤ 0.05) or trend (*p* ≤ 0.10) was identified. Data are presented as mean ± 95% confidence interval (CI), unless otherwise noted. Cohen’s *d* effect sizes were calculated and defined as trivial (0.00–0.19), small (0.20–0.49), medium (0.50–0.79), and large (≥0.80) [22].

Bivariate (Pearson) correlations were computed to determine significant relationships among variables of interest within pooled data. Alpha was set at *p* ≤ 0.05 for statistical significance. All analyses were conducted using the Statistical Package for the Social Sciences (SPSS, Version 21.0; SPSS Inc., Armonk, NY, USA). 

## 3. Results

Table 1 shows the physical characteristics of all subjects. Men were significantly taller and heavier with a greater FFM and FFMI. Further, men had a significantly higher 1-RM CDL and 1-RM CDL: BM ratio. Women had significantly higher %BF (23.3 ± 9.2%) than men (14.8 ± 7.1%). 

Absolute power and velocity across the different loads are presented in Table 2. A significant main effect for load and sex was observed for average power output. However, no significant interaction was observed. When collapsed by sex, average power output was greatest at 60% 1-RM, with no difference observed between 30% and 90% 1-RM. Post hoc analysis on the main effect of sex demonstrated that men produced greater average power (698 ± 36 W) than women (336 ± 36 W; *p* = 0.001; ES = 3.36, Large) when collapsed across all loads. A significant load by sex interaction was observed in peak power (*p* = 0.016), average (*p* = 0.002), and peak (*p* = 0.001) velocity. Men produced significantly higher peak power output across all loads (*p* < 0.001; ES = 3.49, Large). Both men and women produced the highest peak power output at 60% 1-RM, with no difference between 30% and 90% 1-RM. Average velocity declined with the increase in load in both men and women with a higher average velocity observed at 30% 1-RM in men (*p* < 0.001; ES = 2.16, Large). Peak velocity declined in both men and women in a similar manner to average velocity with increasing loads. However, peak velocity was higher at both 30% (*p* = 0.002; ES = 1.80, Large) and 60% 1-RM (*p* = 0.052; ES = 0.94, Large) in men.

Power and velocity data normalized to FFM are presented in Table 3. The greatest average and peak power output was again achieved at 60% 1-RM, irrespective of sex. Further, a main effect of sex was observed for average power (*p* = 0.003) and peak power (*p* = 0.006) with no interaction with load (*p* > 0.05). Men produced significantly greater average power per kg of FFM (10 ± 0 W·kg·FFM^−1^) compared to women (7 ± 0 W·kg·FFM^−1^; *p* = 0.003; ES = 2.01, Large) when collapsed across all loads. A similar relationship was observed when examining peak power (men, 14 ± 1 W·kg·FFM^−1^; women, 11 ± 1 W·kg·FFM^−1^; *p* = 0.006; ES = 1.00, Large). As with absolute values the average and peak velocity both declined with increasing loads. However, post hoc analysis on the main effect of sex showed that women produced significantly higher relative average velocity (0.014 ± 0.001 m·s^−1^·kg·FFM^−1^; *p* = 0.003; ES = 1.33, Large) and peak velocity (0.021 ± 0.001 m·s^−1^·kg·FFM^−1^; *p* = 0.004; ES = 2.00, Large) when compared to men (0.010 ± 0.001 m·s^−1^·kg·FFM^−1^ and 0.015 ± 0.001 m·s^−1^·kg·FFM^−1^, respectively) when collapsed across all loads. No significant interaction was observed.

Power and velocity data normalized to the FFMI are presented in Table 4. Again, the greatest average and peak power were achieved at 60% 1-RM, irrespective of sex. Men produced higher average and peak power across all loads, as evidenced by the main effect of sex (*p* = 0.001) and no interaction (*p* > 0.05). However, the interaction between load and sex approached significance in peak power (*p* = 0.06). Post hoc analysis showed that peak power at 60% 1-RM was greater than both 30% and 90% 1-RM in men, while peak power achieved at 60% 1-RM in women was only greater than 30% 1-RM. Both average and peak velocity declined with increasing loads (*p* = 0.001), with no interaction observed. While the main effect of sex only approached significance in both average (*p* = 0.089) and peak velocity (*p* = 0.079).

Bivariate (Pearson) correlations, which were computed on pooled data among FFM, 1-RM, average power, peak power, average velocity and peak velocity, are included in Table 5. A significant strong positive correlation was observed between FFM and CDL 1-RM (*r* = 0.965, *p* < 0.001). Further, each of these was strongly correlated with absolute average power (*r* > 0.80, *p* < 0.001) and absolute peak power (*r* > 0.79, *p* < 0.001) across all loads tested. The FFM and CDL 1-RM were moderately correlated with absolute average (*r* = 0.55, *p* < 0.02) and absolute peak velocity (*r* ≥ 0.53, *p* < 0.03) at 30% 1-RM. Moderate negative correlations were observed between peak velocity at 90% 1-RM and FFM (*r* = −0.48, *p* = 0.043) and 1-RM (*r* = −0.618, *p* = 0.006). 

## 4. Discussion

We examined sex differences in average power, peak power, average velocity, and peak velocity across 30%, 60%, and 90% 1-RM in the CDL. Our main finding was that men produce significantly higher absolute power across all loads during the performance of the CDL. These higher power outputs appear to be related to the greater muscle mass and strength, as significant correlations were observed between both FFM and strength (1-RM) and power across all loads. Interestingly, absolute peak velocity was only higher in men at lower intensities (i.e., 30%, 60% 1-RM) and when normalized to FFM, women produced significantly higher velocities as evidenced by a statistically significant effect of sex. 

Although the exercise selection of the CDL was novel, the finding of higher absolute power in men was not unexpected. In fact, this has been reported in both trained and untrained populations performing other lifting exercises [9,11,23]. In a population of similar training experience, Thomas et al. [9] reported significantly higher power outputs in men performing upper and lower body exercises across a range of loads (30%–70% 1-RM). Thomas et al. [9] also reported differences between sexes in the optimal load in the squat jump (men: 30%–40% 1-RM; women: 30%–50% 1-RM) and bench press throw (men: 30% 1-RM; women 30%–50% 1-RM). Further, the optimal load for all of the exercises tested occurred in the range of 30%–60% 1-RM [9]. Identification of the optimal load was not the purpose of the current study; however, results suggest that the optimal load for the CDL may be approximately 60% 1-RM since this load resulted in the greatest power output, irrespective of sex.

When normalized to FFM, power was higher across all loads for men. Interestingly, when the relationship between FFM and power was examined, significant positive correlations were observed. A similar relationship was observed between strength (1-RM) and power. Thus, these data seem to suggest that the higher absolute power output and greater strength observed in men is primarily a result of their larger muscle mass. Given the larger FFM observed in males in the current study, the finding of greater strength in men is not surprising. Despite similar rates of protein synthesis following resistance training [24], when compared to women of comparable training status, men are routinely reported to have larger muscle mass [23,25,26]. This difference has been attributed to greater strength levels, which is supported by reported significant positive correlations between FFM and strength in the current study as well as from previous research [25,26,27,28]. However, when normalized to FFM, comparisons of strength have differed. Miller et al. [25] reported significantly greater strength in both upper and lower limbs for men when expressed relative to FFM. Those results are in contrast to Levine et al. [29] in which no sex difference was reported in lower body strength when normalized to FFM. In the current study, men had significantly greater strength when normalized to FFM and were 1.3 times as strong when compared to the women subjects. 

Differences in composition of the FFM between sexes may serve in part to explain the differences observed in this study. We determined body composition through air displacement plethysmography, a two-compartment model in which a constant density is assumed for fat mass and FFM [30]. However, sex differences have been shown to exist in bone and muscle when examining the cross-sectional area of upper and lower limbs via dual energy X-ray absorptiometry (DEXA), a three-compartment model of composition analysis [31]. Further, Miller et al. [25] reported a greater proportion of intramuscular non-contractile tissue in muscle biopsy samples obtained from muscles of the upper and lower body in women when comparing strength and muscle characteristics between sexes. Women had approximately 3.5% greater non-contractile tissue in the biceps brachii, although not statistically significant, and 3.8% greater contractile tissue in the vastus lateralis, which was statistically significant and in support of previous findings [32,33]. Given that non-contractile tissue lacks the ability to generate force; this may contribute somewhat to the differences observed in this study, but certainly does not explain them completely. 

A novel aspect of the current study was the examination of velocity over the range of the loads tested. In contrast to that which was observed in average and peak power, men did not produce significantly higher velocities across all intensities. As expected, velocity decreased as the load increased in both sexes, but absolute velocity was significantly higher only at 30% 1-RM in men, after which there were no significant differences between sexes. Of greater interest, is the fact that only a moderate positive correlation was observed with both FFM and 1-RM at 30%, while a strong negative correlation was observed with both at 90% 1-RM. Further, when normalized to FFM, women produced significantly higher velocities across all loads. A similar result was observed when velocity was examined in relation to FFMI, though this only approached significance. Thus, the current data seem to suggest that the greater power observed in men across all loads may be primarily the result of larger muscle mass and strength. 

A mixed model approach to power training, which incorporates exercises performed with low loads at high velocities and high loads at low velocities and thereby affects a greater portion of the force-velocity relationship, has been suggested as the most efficacious [3,4]. Thus, based upon the data presented herein, to improve power output in men and women strength athletes, practitioners should focus upon those respective areas of weakness. A greater proportion of high velocity-low load training should be incorporated into programs developed for men to improve velocity of movement; while when training women for improved power, more time should be spent improving overall strength with high loads. In support, Cormie et al. [13] reported no significant difference in improvements in jump and sprint performance following either strength or power training in men identified as relatively weak. Further, a larger improvement in strength was observed in those performing strength training compared to power training. 

We recognize the heterogeneity of the athlete population as well as the differing stages of training in which the subjects were involved may serve as a limitation in the current study. While all subjects were currently strength training and had more than a year experience performing the CDL, the cohort included trained Olympic lifters, power lifters, and other strength athletes, who were following sport-specific training regimens and involved in regular activities with definite neuromuscular demands. Thus, results may vary in untrained individuals or in athletes from other sports. However, larger muscle mass [23,25,26,27,28], greater strength [26,31], and power [9,11,23] have been reported in both trained and untrained population samples of men. The results presented herein agree with Garhammer [11] as while men produced significantly higher absolute and relative power over the range of loads lifted, women had higher velocities across all loads when normalized to FFM. If these differences would exist in a population in which men did not have greater strength is unknown. As mentioned previously the effect of gender on loads and power has been understudied. Future research is warranted in this area in which athletes have the same maximal strength levels. 

## 5. Conclusions

This study suggests that the greater power observed across loads in the CDL in men is likely the result of larger muscle mass, which contributes to greater strength levels. In terms of velocity, though men produce significantly higher absolute velocity at 30% 1-RM in the current study, women produce higher velocities across all loads when normalized to FFM. These data support the need to properly assess deficiencies in athletes in order to appropriately develop mixed model training programs to enhance muscular power. 

## Figures and Tables

**Table 1:** Physical characteristics of subjects.

Characteristic	Men (*n* = 9)	Women (*n* = 9)	*p* Value
Age (year)	29 ± 8	29 ± 4.7	0.917
Height (cm)	175.6 ± 5.3	162.3 ± 5.5 *	0.001
Body Mass (kg)	85.5 ± 16.2	62.0 ± 7.3 *	0.002
%BF	14.8 ± 7.1	23.3 ± 9.2 *	0.042
FFM (kg)	72.0 ± 8.9	47.1 ± 3.9 *	0.001
FFMI	23.3 ± 1.7	17.9 ± 1.4	0.001
1-RM CDL (kg)	197.8 ± 46.3	100.0 ± 18.4 *	0.001
1-RM CDL: BM ratio	2.3 ± 0.3	1.6 ± 0.4 *	0.001
1-RM CDL: FFM ratio	2.7 ± 0.4	2.1 ± 0.3 *	0.002

Data are mean ± SD. BF, body fat; FFMI, fat free mass index; 1-RM, one-repetition maximum; CDL, conventional deadlift; BM, body mass; FFM, fat free mass; * Significant difference between sexes.

**Table 2:** Absolute power and velocity for men and women during CDL exercise at varying loads.

Variable	Men	Women	Combined	*p* Value
**Average Power (W)**				
30% 1-RM	626 (519, 733)	299 (192, 407)	463 (387, 539)	L = 0.001
60% 1-RM	822 (719, 925)	388 (285, 490)	605 (532, 678) ^†^	S = 0.001 ^#^
90% 1-RM	646 (590, 702)	320 (264, 377)	483 (444, 523) ^‡^	L × S = 0.134
**Peak Power (W)**				
30% 1-RM	919 (759, 1079) *	442 (282, 602)	681 (567, 793)	L = 0.001
60% 1-RM	1247 (1097, 1396) ^†,^*	569 (419, 718) ^†^	908 (802, 1013)	S = 0.001
90% 1-RM	927 (845, 1009) ^‡,^*	541 (459, 623)	734 (676, 792)	L × S = 0.016
**Average Velocity (m·s^−1^)**				
30% 1-RM	1.09 (1.02, 1.15) *	0.89 (0.82, 0.96)	0.99 (0.94, 1.04)	L = 0.001
60% 1-RM	0.73 (0.67, 0.79) ^†^	0.67 (0.61, 0.73) ^†^	0.70 (0.65, 0.74)	S = 0.001
90% 1-RM	0.38 (0.33, 0.45) ^†,‡^	0.38 (0.32, 0.44) ^†,‡^	0.38 (0.34, 0.43)	L × S = 0.002
**Peak Velocity (m·s^−1^)**				
30% 1-RM	1.59 (1.48, 1.70) *	1.32 (1.21, 1.42)	1.45 (1.38, 1.53)	L = 0.001
60% 1-RM	1.10 (1.01, 1.19) ^†,^*	0.98 (0.89, 1.07) ^†^	1.04 (0.98, 1.10)	S = 0.052
90% 1-RM	0.57 (0.45, 0.68) ^†,‡^	0.63 (0.52, 0.75) ^†,‡^	0.60 (0.52, 0.68)	L × S = 0.001

Data are mean ± 95% confidence interval. Combined data are collapsed by sex; L = effect of load; S = effect of sex; L × S = interaction effect; ^†^ Significantly different from 30% 1-RM; ^‡^ Significantly different from 60% 1-RM; ^#^ Main effect of sex; * Significant difference between sexes at specific load.

**Table 3:** Power and velocity normalized to FFM for men and women during CDL exercise at varying loads.

Variable	Men	Women	Combined	*p* Value	
**Average Power (W·kg·FFM^−1^)**					
30% 1-RM	9 (6, 11)	6 (4, 8)	8 (6, 9)	L = 0.001	
60% 1-RM	11 (10, 13)	8 (7, 10)	10 (9, 11) ^†^	S = 0.003 ^#^	
90% 1-RM	9 (8, 10)	7 (6, 8)	8 (7, 8) ^‡^	L × S = 0.618	
**Peak Power (W·kg·FFM^−1^)**					
30% 1-RM	13 (10, 16)	10 (7, 12)	11 (9, 13)	L = 0.001	
60% 1-RM	17 (15, 19)	12 (10, 14)	15 (13, 16) ^†^	S = 0.006 ^#^	
90% 1-RM	13 (11, 14)	12 (10, 13)	12 (11, 13) ^‡^	L × S = 0.121	
**Average Velocity (m·s^−1^·kg·FFM^−1^)**					
30% 1-RM	0.015 (0.013, 0.017)	0.019 (0.017, 0.021)	0.017 (0.016, 0.019)	L = 0.001	
60% 1-RM	0.010 (0.009, 0.012)	0.014 (0.013, 0.016)	0.012 (0.011, 0.013) ^†^	S = 0.003 ^#^	
90% 1-RM	0.006 (0.004, 0.007)	0.008 (0.007, 0.010)	0.007 (0.006,0.008) ^†,‡^	L × S = 0.279	
**Peak Velocity (m·s^−1^·kg·FFM^−1^)**					
30% 1-RM	0.022 (0.019, 0.026)	0.028 (0.025, 0.031)	0.025 (0.023, 0.028)	L = 0.001	
60% 1-RM	0.016 (0.013, 0.018)	0.021 (0.019, 0.023)	0.018 (0.017, 0.020) ^†^	S = 0.004 ^#^	
90% 1-RM	0.008 (0.006, 0.011)	0.014 (0.011, 0.016)	0.011 (0.009, 0.013) ^†,‡^	L × S = 0.973	

Data are mean ± 95% confidence interval. L = effect of load; S = effect of sex; L × S = interaction effect; ^†^ Significantly different than 30% 1RM; ^‡^ Significantly different than 60% 1RM; ^#^ Main effect of sex.

**Table 4:** Power and velocity normalized to FFMI for men and women during CDL exercise at varying loads.

Variable	Men	Women	Combined	*p* Value
**Average Power (W·FFMI^−1^)**				
30% 1-RM	27 (22, 32)	17 (12, 22)	22 (18, 25)	L = 0.001
60% 1-RM	35 (31, 39)	22 (18, 26]	28 (25, 31) ^†^	S = 0.001 ^#^
90% 1-RM	28 (25, 30)	18 (16, 20]	23 (21, 25) ^‡^	L × S = 0.400
**Peak Power (W·FFMI^−1^)**				
30% 1-RM	39 (32, 47)	25 (17, 32)	32 (27, 37)	L = 0.001
60% 1-RM	53 (47, 59) ^†^	32 (26, 38) ^†^	42 (38, 47) ^†^	S = 0.001 ^#^
90% 1-RM	40 (36, 44) ^‡^	30 (26, 34)	35 (32, 38) ^‡^	L × S = 0.060
**Average Velocity (m·s^−1^·FFMI^−1^)**				
30% 1-RM	0.047 (0.042, 0.052)	0.050 (0.045, 0.055)	0.049 (0.045, 0.052)	L = 0.001
60% 1-RM	0.031 (0.027, 0.035)	0.038 (0.034, 0.041)	0.034 (0.032, 0.037) ^†^	S = 0.089
90% 1-RM	0.017 (0.013, 0.021)	0.021 (0.017, 0.025)	0.019 (0.016, 0.022) ^†,‡^	L × S = 0.479
**Peak Velocity (m·s^−1^·FFMI^−1^)**				
30% 1-RM	0.069 (0.061, 0.077)	0.074 (0.066, 0.082)	0.071 (0.066, 0.077)	L = 0.001
60% 1-RM	0.048 (0.041, 0.054)	0.055 (0.049, 0.061)	0.051 (0.047, 0.056) ^†^	S = 0.079
90% 1-RM	0.025 (0.018, 0.032)	0.036 (0.029, 0.043)	0.030 (0.025, 0.036) ^†,‡^	L × S = 0.312

Data are mean ± 95% confidence interval. L = effect of load; S = effect of sex; L × S = interaction effect; ^†^ Significantly different than 30% 1-RM; ^‡^ Significantly different than 60% 1-RM; ^#^ Main effect of sex.

**Table 5:** Bivariate correlations between FFM, strength (1-RM), absolute measures of power and velocity.

Variable	Value	FFM	CDL 1-RM
Fat Free Mass (FFM)	*r*		0.965
	*p*		0.000 *
CDL 1-RM	*r*	0.965	
	*p*	0.000 *	
Average Power 30% 1-RM	*r*	0.811	0.892
	*p*	0.000 *	0.000 *
Average Power 60% 1-RM	*r*	0.931	0.958
	*p*	0.000 *	0.000 *
Average Power 90% 1-RM	*r*	0.911	0.870
	*p*	0.000 *	0.000 *
Peak Power 30% 1-RM	*r*	0.811	0.898
	*p*	0.000 *	0.000 *
Peak Power 60% 1-RM	*r*	0.938	0.960
	*p*	0.000 *	0.000 *
Peak Power 90% 1-RM	*r*	0.835	0.782
	*p*	0.000 *	0.000 *
Average Velocity 30% 1-RM	*r*	0.558	0.554
	*p*	0.016 *	0.017 *
Average Velocity 60% 1-RM	*r*	0.311	0.321
	*p*	0.209	0.194
Average Velocity 90% 1-RM	*r*	−0.229	−0.392
	*p*	0.631	0.107
Peak Velocity 30% 1-RM	*r*	0.526	0.545
	*p*	0.025 *	0.019 *
Peak Velocity 60% 1-RM	*r*	0.389	0.382
	*p*	0.111	0.118
Peak Velocity 90% 1-RM	*r*	−0.481	−0.618
	*p*	0.043 *	0.006 *

*r* represents Pearson correlations; *p* values represent 2-tailed testing; * significant at *p* ≤ 0.05.
